# Adipoclast: a multinucleated fat-eating macrophage

**DOI:** 10.1186/s12915-021-01181-3

**Published:** 2021-11-19

**Authors:** Antoni Olona, Subhankar Mukhopadhyay, Charlotte Hateley, Fernando O. Martinez, Siamon Gordon, Jacques Behmoaras

**Affiliations:** 1 Department of Infectious Disease, Imperial College London, Hammersmith Hospital, London, UK; 2 MRC Centre for Transplantation, Peter Gorer Department of Immunobiology, School of Immunology and Microbial Sciences, King’s College London, Great Maze Pond, London, SE1 9RT UK; 3 Centre for Inflammatory Disease, Imperial College London, Hammersmith Hospital, London, UK; 4 Faculty of Health and Medical Sciences, University of Surrey, Guildford, UK; 5 Graduate Institute of Biomedical Sciences, College of Medicine, Chang Gung University, Taoyuan City, Taiwan; 6 Sir William Dunn School of Pathology, University of Oxford, Oxford, UK; 7 Center for Computational Biology and Program in Cardiovascular and Metabolic Disorders, Duke-NUS Medical School, Singapore, Singapore

## Abstract

Cell membrane fusion and multinucleation in macrophages are associated with physiologic homeostasis as well as disease. Osteoclasts are multinucleated macrophages that resorb bone through increased metabolic activity resulting from cell fusion. Fusion of macrophages also generates multinucleated giant cells (MGCs) in white adipose tissue (WAT) of obese individuals. For years, our knowledge of MGCs in WAT has been limited to their description as part of crown-like structures (CLS) surrounding damaged adipocytes. However, recent evidence indicates that these cells can phagocytose oversized lipid remnants, suggesting that, as in osteoclasts, cell fusion and multinucleation are required for specialized catabolic functions. We thus reason that WAT MGCs can be viewed as functionally analogous to osteoclasts and refer to them in this article as adipoclasts. We first review current knowledge on adipoclasts and their described functions. In view of recent advances in single cell genomics, we describe WAT macrophages from a ‘fusion perspective’ and speculate on the ontogeny of adipoclasts. Specifically, we highlight the role of CD9 and TREM2, two plasma membrane markers of lipid-associated macrophages in WAT, which have been previously described as regulators of fusion and multinucleation in osteoclasts and MGCs. Finally, we consider whether strategies aiming to target WAT macrophages can be more selectively directed against adipoclasts.

Macrophages have a unique potential to fuse with themselves to form multinucleated giant cells (MGCs) [1]. During homeostasis, the majority of macrophages fuse infrequently and reside in tissues as mononuclear cells. The exception to this rule is the osteoclast of bone, a multinucleated monocyte/macrophage [2] that originated from embryonic erythro-myeloid progenitors and is responsible for the resorption of mineralized bone [3]. The multinucleation capability of the osteoclast correlates with its resorptive activity, suggesting that cell fusion confers a specialized stage of differentiation lacking in the mononuclear state [1]. The concept of a cellular gain of function as a result of fusion/multinucleation is supported by a recent discovery showing that multinucleated osteoclasts can undergo fission to form osteomorphs, daughter cells transcriptionally distinct from osteoclasts [4]. While osteoclasts regulate bone mass, pathological macrophage fusion can be an immune response to infectious pathogens (e.g. *Mycobacterium tuberculosis*) or foreign materials. MGCs are derived from monocyte progenitors [4] but their precise role within the granuloma is not yet clear. On the other hand, foreign-body giant cells (FBGCs) can be involved in the uptake of larger particles [5], an observation confirmed in vitro [6]. These observations suggest that enhanced phagocytic clearance of large particulates is an adaptive phenomenon resulting from macrophage fusion and multinucleation.

The adipose tissue contains macrophages and during obesity, their number increases significantly (up to 50% of all cells) to correlate with metabolic dysfunction characterized by inflammation, fibrosis and insulin resistance [7–11]. Their histological description as crown-like structures (CLS) refers to MGCs associated with necrotic adipocytes [12]; however, recent evidence demonstrated that these MGCs can phagocytose lipid remnants more efficiently when compared to unfused WAT macrophages [13].

There is an intriguing association between lipids and macrophage fusion. Cholesterol-rich MGCs have been reported as a frequent and non-specific histological feature in lung biopsies [14]. Historically, the Touton giant cell, which is frequently found in lesions containing high levels of cholesterol and lipid deposits, has been described as a product of fusion between macrophage-derived foam cells [15]. Multinucleated foam cells have been indeed observed as a result of high-fat diet in inflammatory sites such as the synovium [16]. Recent evidence shows that common monocyte progenitors accumulate cholesterol and lipids, which are required for MGC formation [17]. These studies suggest that a lipid rich microenvironment such as the white adipose tissue (WAT) can be ‘fusogenic’ for resident macrophages. Based on recent findings published by Braune and colleagues [13], and the existing literature on osteoclasts and MGCs, we postulate that macrophage fusion and multinucleation in the WAT may initiate a ‘gain of function’ to clear increasingly stressed adipocytes under metabolically challenging conditions such as obesity. Thus, in this review, we refer to MGCs of crown-like structures (CLS) as adipoclasts, the ‘fat-resorbing osteoclasts’ of the white adipose tissue (Fig. 1A, B). The term adipoclast does not differentiate between the MGCs with different nuclei numbers (binuclear, 2–4, > 4) and differs from the designation lipid-associated macrophage (LAM) by its unique multinucleated feature. The choice of this term is based on the (i) wide description of the CLS histologically in the white adipose tissue, (ii) their recent functional annotation as catabolic cells following fusion/multinucleation [13], and (iii) the functional analogy with osteoclasts—hence the suffix ‘clast’.

**Fig. 1:**
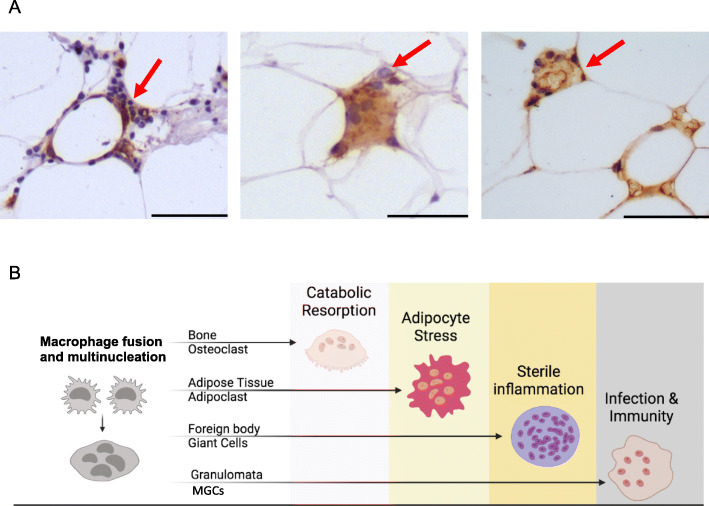
**A** Representative CD68 immunohistochemistry (brown) showing multinucleated adipoclasts (red arrows) in the white adipose tissue of obese patients undergoing bariatric surgery; scale bar, 50μm. **B** Macrophage fusion and multinucleation in health and disease. In addition to osteoclasts, foreign body giant cells and MGCs, adipoclasts contribute to the clearance of stressed adipocytes in the white adipose tissue

We first describe the current knowledge on CLS and their proposed function. We then review recent advances in WAT single cell transcriptomics, with a specific focus on TREM2 and CD9, membrane receptors that have been previously described in macrophage fusion and multinucleation. We highlight the respective roles of TREM2 and CD9 in osteoclasts, in order to speculate on the adipoclasts’ origin and function. Finally, we discuss whether recent macrophage-targeting therapies in the fat may be beneficial or fine-tuned in targeting adipoclasts in obesity. The review does not cover the polarization of macrophages in adipose tissue nor the significance of WAT inflammation in insulin resistance and metabolic disorders in general—an area that is amply covered by excellent reviews (some examples include [10, 18–23])

## Crown-like structures are adipoclasts

The infiltration of immune cells in the obese adipose tissue was shown in the 1960s [24, 25] and then overlooked for almost four decades, except for an in vitro study showing that insulin resistance in adipocytes can be caused by a macrophage-derived mediator [26]. The presence of macrophages in human and mice adipose tissue was shown by several groups and while some reported their tissue localization adjacent to adipocytes, others highlighted their morphological appearance as MGCs arising from cell fusion [11, 12, 27, 28]. Clement et al. isolated CD14+ cells from the stromal vascular fraction (SVF) of human subcutaneous WAT, using CD14-coupled magnetic microbeads and confirmed the presence of macrophages in adipose tissue by immunohistochemistry [27]. Two contemporaneous studies reported the existence of macrophage syncytia (or MGCs) in the WAT of genetically obese mice (*ob/ob*) [11, 28]. From a histological point of view, Cinti et al. were the first to designate the WAT multinucleated macrophages as crown-like structures (CLS) [12], surrounding necrotic or lipolytic adipocytes [12, 28]. Today it is well-established that adipose tissue CLS contain multinucleated macrophages (i.e. adipoclasts; Fig. 1A) and increase in frequency with obesity. The origin of this augmented macrophage infiltration in the WAT is thought to be blood monocytes [29] and the literature on CLS has long assumed that these cells are implicated in efferocytosis of dead adipocytes because of their histological localization around dead adipocytes. A recent study brought definitive evidence by live imaging the WAT MGCs (i.e. adipoclasts) in mice, showing that these cells can take up lipid remnants which were not ingestible by mononuclear macrophages in the WAT [13]. A bead phagocytosis assay confirmed these findings and showed that, like MGCs [6], adipoclasts can phagocytose large particles [13]. Interestingly, confirming the previous associations between MGCs and lipids, adipoclasts display a relatively high lipid content [13] and this is not surprising given the fusogenic properties of the long-chain fatty acid binding scavenger receptor CD36 in macrophages [30].

In summary, while it is well-accepted that adipoclasts are specialized in efferocytosis of damaged adipocytes, many questions remain regarding the mechanisms underlying this process, as well as the other advantages that cell fusion and multinucleation may confer in the context of prolonged obesity. Furthermore, given the presence of mononucleated, often foamy macrophages in WAT, it is necessary to consider more trophic functions and crosstalk between macrophages and adipocytes [31] including the role of CD36 and other macrophage scavenger receptors [32], as well as clearance functions.

## The complexity behind adipoclast function

During prolonged obesity, adipose tissue remodelling is a well-described phenomenon that consists in depot-dependent adipocyte death associated with macrophage infiltration [33, 34]. Our limited understanding of adipoclast function is due to the complex aspects of the evolution of adipocyte cell state under metabolically impaired conditions (see review [35]). During obesity, adipocytes can undergo various forms of death [36]—apoptotic [37], necrotic [12], and pyroptotic [38]. In addition, pre-adipocytes (i.e. the precursor of adipocytes) have been described to undergo senescence through different mechanisms during obesity [39, 40]. On the other hand, the macrophage clearance mechanisms of damaged adipocytes were reported to be through lysosomal exocytosis [41], in addition to phagocytosis [13]. By live-imaging, a recent report showed the requirement of a size threshold for efferocytosis of lipid remnants [42]. Adipocyte death induces a metabolically activated and pro-inflammatory macrophage phenotype [42]. Paradoxically, the clearance of dead adipocytes by CLS was also linked to preadipocyte proliferation [43], suggesting an adipogenic role for adipoclasts. Adding to this complexity, different fat depots (visceral vs. subcutaneous) can display different prevalence in adipocyte cell death. It was reported that CLS were widespread in visceral compared with subcutaneous fat in genetically obese mice (db/db and ob/ob) [44]. In keeping with this, adipoclast infiltrates may differ between murine and human WAT. In mice, a prolonged high-fat diet of 24 weeks is required to observe the adipoclasts histologically [13], suggesting that prolonged obesity is a prerequisite for multinucleation of these cells.

Hence adipoclasts have been linked to adipocytes in different cellular states that describe broadly cellular stress and ultimately death. This raises the question of whether adipoclasts can ‘sense’ a particular adipocyte state and whether their fusion from mononuclear macrophages is triggered through adipocyte-derived markers of stress. For instance, using a co-culture setup, it was shown that adipocyte death triggers MGC formation in vitro [13]. Further experiments will be crucial in order to establish the exact mechanisms underlying this process.

## Adipoclasts and/or their precursors display multinucleation markers

While it is accepted that obesity is associated with a shift toward pro-inflammatory macrophage function [45–47], WAT macrophages have a unique polarization state (metabolically activated macrophages [48]) and paradoxically, crown-like structures contain the M2-like marker CD206 (mannose receptor) and CD11c expressing macrophages [49]. Recent single cell transcriptomics studies revealed the different subtypes of adipose tissue macrophages and their evolution upon obesogenic conditions [50–54]. Two markers of white adipose tissue macrophages of particular interest include TREM2 and CD9. Jaitin et al. were first to describe a TREM2-expressing lipid-associated macrophage (LAM) subset in human WAT [53], later confirmed by a separate study [51]. Similarly, CD9, another marker of LAMs [53], was found to colocalize with the pan-macrophage marker CD68 in human WAT [54]. Notably, TREM2+ and CD9+ LAMs were found to be part of CLS [52, 53] and their frequency increased with obesity in mice and humans [50, 51] with a shift toward a pro-inflammatory polarization characterized by IL-1β and TNF production [51].

None of the single cell RNA-seq studies in the WAT distinguished multinucleated macrophages (i.e. adipoclasts) from other macrophage subsets. Although technically challenging, this could have been attempted by sorting LAMs with > 2 nuclei. The advantage of such an approach would have been the identification of potential precursors of adipoclasts, in order to make a distinction between ‘fusion-competent’ LAMs and adipoclasts, as well as the polarization state of each cell type. Nevertheless, the recent single cell transcriptomic approaches in human WAT suggest that adipoclasts and/or adipoclast precursors express TREM2 and CD9 [51–54].

## TREM2 and CD9: a parallel between adipoclasts and osteoclasts

The existence of CD9+ and TREM2+ adipoclasts is worth highlighting from a macrophage fusion perspective, especially given the relevance of these two membrane proteins in osteoclast and MGC fusion and multinucleation.

Besides its widely studied role in microglial phagocytosis [55] and neurodegeneration [56, 57], *TREM2* (the triggering receptor expressed on myeloid cells 2) is essential for macrophage multinucleation as part of a signalling pathway that includes DAP12 and Syk [58]. TREM2 regulates osteoclast formation [59–61] and a recent report shows its regulatory role in granuloma formation through recruitment of mycobacterium-permissive macrophages [62]. Furthermore, deletions or loss-of-function mutations in either DAP12 or *TREM2* are causally associated with Nasu–Hakola disease, a dementia associated with bone cystic lesions [63, 64]. Importantly, mutations in *TREM2* and *DAP12* induce defective multinucleation in osteoclasts, resulting in impaired bone resorption [60]. *Trem2* is a *trans*-acting genetic regulator of a macrophage multinucleation gene co-expression network [65, 66], which also includes genes belonging to the Pi3K-mTORC1 pathway that controls osteoclast multinucleation and bone mass [66]. The TREM2-PI3K-mTOR axis is indeed well-defined in microglia [67] and the activation of PI3K signalling is a common feature of osteoclasts and MGCs [58, 68].

Jaitin et al. identified TREM2, not only as a marker, but also as a driver of the LAM cell molecular program as lipid uptake and storage were abrogated in the absence of *Trem2* [53]. Interestingly, apolipoprotein E (*ApoE*) is a *Trem2* ligand [69, 70] and both *Trem2* and *ApoE* are expressed by a subpopulation of tumour-associated macrophages [71]. Macrophages can fuse with tumour cells and contribute to tumour heterogeneity [72], but a potential role of *Trem2* in this process is yet to be found. The lipid sensing role of *TREM2* has been shown as part of the microglia response [73] but also during infection, as TREM2 is capable of recognizing mycobacterial cell-wall mycolic acid (MA)-containing lipids [62]. This raises the possibility of a lipid uptake through TREM2 that can be a prerequisite mechanism for macrophage fusion and multinucleation. Local lipid changes are principal regulators of adipose tissue macrophage recruitment [74]. Interestingly, single cell RNA-sequencing analysis of aortic CD45+ cells from atherosclerotic high-fat diet-fed (*Ldlr*^-/-^) mice identified macrophages with high *Trem2* expression, specialized in lipid metabolism/catabolism and enriched in the osteoclast gene signature [75]. If one extrapolates these findings to the WAT, it is plausible that *Trem2* expressing macrophages accumulate lipids and become fusogenic, giving rise to adipoclast precursors and adipoclasts. Fusion and multinucleation could be considered as the final differentiation step of these precursors. However, the exact *Trem2*-dependent and lipid-related mechanisms allowing the transition from fusion-competent adipoclast precursors to adipoclasts remain to be identified, and in that sense, some parallels drawn from knowledge on osteoclast lipid metabolism may be of relevance. Cholesterol is indispensable for membrane fusion and osteoclast v-ATPase activity [76, 77] and *Ldlr*^-/-^ mice have defective osteoclast fusion [78]. Since osteoclast formation, survival and morphology are highly dependent on exogenous cholesterol/lipoproteins [79], adipoclast integrity and function may also be under the influence of a cholesterol-rich environment in the adipose tissue. Similarly, saturated fatty acids enhance osteoclast survival [80] and palmitic acid increases RANKL-mediated osteoclast differentiation [81]. On the other hand, short-chain fatty acids such as propionate and butyrate induce metabolic reprogramming of osteoclasts and downregulate essential osteoclast genes [82]. This suggests that individual lipid species may have opposing roles on osteoclast differentiation and fusion and therefore the lipid dynamics in the WAT during obesity may determine the formation of adipoclasts. In this regard, it has been shown that ablation of fat cells in adult mice can induce massive bone gain [83]. As the diet and microbiome significantly contribute to the reserve and processing of fatty acids, the lipid composition of WAT under obesogenic conditions [84] can be a pivotal factor in determining adipoclast formation and function.

Tetraspanins are a superfamily of membrane proteins, and among them, CD9 and CD81 are closely related and known to control cell–cell fusion as they negatively regulate fusion of osteoclasts and MGCs [85, 86]. These proteins facilitate the organization of integrins and influence macrophage motility [87]. CD9/CD81 double-null mice spontaneously develop MGCs in the lung, showing enhanced osteoclastogenesis in the bone and signs of accelerated ageing with atrophy of adipose tissue [86, 88]. Interestingly, while CD9 has been robustly linked to WAT macrophages [51, 53, 54], CD81 has been recently described as a beige adipocyte progenitor cell marker and regulator of de novo beige fat biogenesis following cold exposure [89]. The potential involvement of CD81 in adipoclast differentiation and function remains to be identified. Given that CLS have been described to be an adipogenic niche for adipocyte progenitor cells [43], CD81 may be involved in a possible adipocyte progenitor-adipoclast/adipoclast-precursor interaction. Notably, tetraspanins are the only inhibitors of fusion that have been so far identified. Because their downregulation induces membrane fusion [1, 90], CD9 and CD81 may be expressed in adipoclast precursors and undergo down-regulation when fusion occurs. Hence, the transcriptomic characterization of CD9+ mononucleated and multinucleated cells in the WAT can confirm the precise role of tetraspanins in adipoclast formation.

**Fig. 2:**
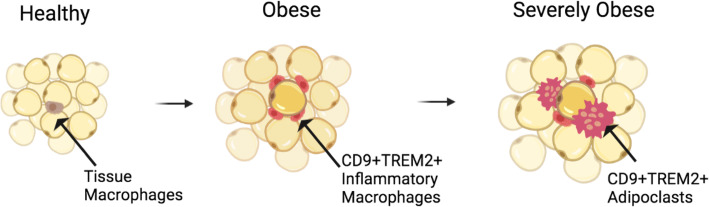
The transition from obese to severely obese state is characterized by increased macrophage infiltration and the formation of TREM2 and CD9 expressing pro-inflammatory macrophages that eventually give rise to multinucleated adipoclasts surrounding stressed adipocytes. How fusion/multinucleation affects the expression TREM2/CD9 and whether this causes de novo expression of adipoclasts markers is yet to be determined

In summary, the presence of TREM2^+^CD9^+^ adipoclasts or adipoclasts precursors seems to correlate with WAT inflammation and the severity of obesity-related pathologies (Fig. 2). In support of the pathogenic role of adipoclasts, a scar-associated and pro-fibrotic TREM2^+^CD9^+^ subpopulation of macrophages was identified in cirrhotic human liver [91]. These scar-associated macrophages were conserved in mice and express osteopontin (SPP1) [91], a protein that regulates FBGC formation [92] and osteoclast fusion and resorption [93]. Whether the scar-associated macrophages can fuse with each other remains to be confirmed. In non-alcoholic steatohepatitis (NASH), a specific macrophage population is characterized by high levels of expression of *Trem2* [94] and other lipid-associated macrophage markers, forming hepatic CLS [95]. A NASH diet causes a partial loss of Kupffer cell identity, induction of *Trem2* and *Cd9* expression, and cell death in mice [96]. Interestingly, the expression of *Trem2* and *Cd9* is a result of substantial reprogramming of the Kupffer cell regulatory landscape due to the prolonged exposure to the NASH diet [96]. Hence, an interesting parallel can be made with TREM2^+^CD9^+^ adipoclasts, which may form as a result of chronic obesogenic conditions, whereby membrane fusion and multinucleation are likely to induce changes in the transcriptomic/epigenetic landscape, allowing phagocytosis of damaged adipocytes. In addition to metabolic tissues, TREM2^+^CD9^+^ microglia in the brain may play a pathogenic role. It is intriguing that lipid-droplet-accumulating microglia (a subgroup presumably distinct from the disease-associated microglia expressing TREM2 and CD9 [97]), represent a dysfunctional and proinflammatory state in the ageing brain [98].

## Targeting macrophages and/or adipoclasts in obesity?

To date, it is well-accepted that obesity triggers the recruitment of monocytes into adipose tissue to promote inflammation, which itself may cause ectopic fat deposition in the liver and insulin resistance [99, 100]. The discovery of adipose tissue TNF [101, 102] and a decade later the monocyte-chemoattractant protein 1 (MCP-1) [103, 104], proved the importance of WAT inflammation and its indisputable macrophage component in the metabolic syndrome. Logically, this has seen the emergence of macrophage-targeting therapies that were initially aiming to inhibit the recruitment of these cells [105–107]. With the increasing recognition of macrophage metabolism in the regulation of its immune function [108], novel initiatives target mitochondrial function in macrophages [109, 110], given the relevance of mitochondrial oxidative phosphorylation in diet-induced obesity [111]. Drug delivery approaches, including nanomaterial-based ones targeting macrophages, hold promise [112]. Furthermore, in addition to their professional phagocytic activity and plasticity [113], tissue macrophages have unique features that differentiate them from surrounding cells. For instance, their enhanced sensitivity to changes in intracellular potassium levels and inflammasome activation [114], makes them attractive targets for Na^+^/K^+^-ATPase blockers such as ouabain [115]. A recent study exemplifies the strategic relevance of macrophage-targeted pharmacological interventions in obesity: macrophage-derived PDGFcc production is regulated by diet and increases lipid storage by white adipocytes [116].

When considering macrophage-targeted treatments in adipose tissue, it is crucial to keep in mind the heterogeneity and master regulatory role of macrophages in the development and homeostatic function of adipose tissue. It has become evident that macrophages express organ-specific genes in addition to canonical macrophage genes, a phenomenon referred to as niche-specific programming [96, 117]. The recently identified sympathetic neuron-associated macrophages increase with obesity and can be targeted for the browning of white fat [118]. This shows the heterogeneity of adipose tissue macrophages, which should be taken into account in any pharmacological approach aiming to reduce obesity-related complications. During homeostasis, many aspects of the mature function of macrophages are controlled by CSF1 and IL-34, which both bind CSF1R, a receptor restricted to cells of the myeloid lineage. Furthermore, Trib1, an adaptor protein involved in protein degradation, is critical for the differentiation of tissue-resident macrophages [119], while receptors known to be preferentially expressed by mononuclear phagocytes such as TREM2 [55, 120] and MARCO [121, 122], regulate an array of tissue-resident macrophage function including efferocytosis (TREM2) and scavenging (MARCO). The genetic deletion of *Csf1r* in rats and *Trib1* in mice reduces adipose tissue mass [119, 123], while *Trem2*^-/-^ and *Marco*^-/-^ LAMs lose their efficacy in lipid buffering [53, 124]. Of note, CSF1R on microglial cells can control hypothalamic control of energy homeostasis in mice [125, 126] which suggests that CSF1R may be responsible for local and systemic control of adiposity. When considering macrophage-targeted therapies, a possible non-myeloid expression of some markers (e.g. *Trem2*) should be taken into consideration as it may influence metabolic health [127]. Altogether, these studies suggest that healthy macrophage differentiation and function is an unconditional part of adipose tissue homeostasis and therapeutic approaches must differentiate between optimal macrophage presence and pathological infiltration and accumulation of these cells.

Based on current knowledge, adipoclasts are likely to form when relatively high numbers of macrophages infiltrate the adipose tissue due to prolonged obesity. It is still not clear whether adipoclasts are only homokaryons or whether they can also form by fusion of mononucleated macrophages and adipocytes. Here we argue that inhibiting adipoclast formation may improve insulin sensitivity. Rather than global approaches aiming to target adipose tissue macrophages, one can envisage inhibition of adipoclast formation. However, such therapies require a better understanding of adipoclast formation and the identification of novel markers that differentiate mononucleated precursors from multinucleated fused cells. Integrating transcriptomic, epigenetic and metabolic events that accompany cell fusion and multinucleation in the WAT will fine-tune cell-based therapies in obesity and metabolic syndrome.

## Data Availability

Not applicable.
